# PD-L1 is a direct target of cancer-FOXP3 in pancreatic ductal adenocarcinoma (PDAC), and combined immunotherapy with antibodies against PD-L1 and CCL5 is effective in the treatment of PDAC

**DOI:** 10.1038/s41392-020-0144-8

**Published:** 2020-04-17

**Authors:** Xiuchao Wang, Xin Li, Xunbin Wei, Haiping Jiang, Chungen Lan, Shengyu Yang, Han Wang, Yanhui Yang, Caijuan Tian, Zanmei Xu, Jiangyan Zhang, Jihui Hao, He Ren

**Affiliations:** 1 Department of Gastroenterology, Center of Tumor Immunology and Cytotherapy, The Affiliated Hospital of Qingdao University, 266003 Qingdao, China; 2 Department of Cellular and Molecular Physiology, Penn State College of Medicine, Hershey, PA 17033 USA; 3 Department of Pancreatic Cancer, Tianjin Medical University Cancer Institute and Hospital, 300060 Tianjin, China; 4 Department of Geriatric Dentistry, Beijing Laboratory of Biomedical Materials, Peking University School and Hospital of Stomatology, and Biomedical Engineering Department, Peking University, 100081 Beijing, China; 5 Department of Applied Statistics, College of Science, Hebei University of Technology, 300401 Tianjin, China; 6 NHC Key Laboratory of Hormones and Development (Tianjin Medical University), Tianjin Key Laboratory of Metabolic Diseases, Tianjin Medical University Chu Hsien-I Memorial Hospital & Tianjin Institute of Endocrinology, 300134 Tianjin, China; 7 Tianjin Marvel Medical Laboratory, Tianjin Marvelbio Technology Co., Ltd, 300381 Tianjin, China

**Keywords:** Gastrointestinal cancer, Tumour immunology

## Abstract

High expression of PD-L1 marks the poor prognosis of pancreatic ductal adenocarcinomas (PDAC). However, the regulatory mechanism of PD-L1 remains elusive. We recently reported that cancer Forkhead box protein 3 (Cancer-FOXP3 or C-FOXP3) promoted immune evasion of PDAC by recruiting Treg cells into PDAC via upregulation of CCL5. In this study, we confirmed that PD-L1 was overexpressed in PDAC samples from two independent cohorts of patients with radical resection. Moreover, C-FOXP3 was colocalized and correlated with the expression of PD-L1 in tumor cells at the mRNA and protein levels, and this finding was confirmed by the The Cancer Genome Atlas (TCGA) database. Chromatin immunoprecipitation (ChIP) revealed that C-FOXP3 directly bound to the promoter region of PD-L1 in pancreatic cancer cells. Furthermore, overexpression of C-FOXP3 activated the luciferase reporter gene under the control of the PD-L1 promoter. However, mutation of the binding motif-a completely reversed the luciferase activity. In addition, C-FOXP3-induced upregulation of PD-L1 effectively inhibited the activity of CD8^+^ T cells. Based on our recent finding that the CCL-5 antibody achieved a better response to PDAC models with high C-FOXP3 levels, we further demonstrated that the PD-L1 antibody strengthened the antitumor effect of CCL-5 blockade in xenograft and orthotopic mouse models with high C-FOXP3 levels. In conclusion, C-FOXP3 directly activates PD-L1 and represents a core transcription factor that mediates the immune escape of PDAC. Combined blockade of PD-L1 and CCL-5 may provide an effective therapy for patients with PDAC that have high C-FOXP3 levels.

## Introduction

Activation of T lymphocytes and associated cytokines in the body is regulated by the B7:CD28 family of costimulatory molecules.^1^ In many cancer types, T cell activation is inhibited by the interaction of B7-1 with the transmembrane protein programmed death 1 ligand 1 (PD-L1). Upregulation of PD-L1 in a series of human cancers, including non-small cell lung cancer (NSCLC), melanoma and gastric cancer, results in T cell inactivation and escape of these cancers from immune surveillance.^2^ Therefore, therapeutic blockade of PD-L1 is rapidly becoming a therapeutic option for the treatment of cancers that exhibit overexpression of PD-L1. In fact, tumor expression of PD-L1 functions as a marker for predicting response to therapy in certain types of cancer, as exemplified by NSCLC.^3^ It is therefore critical to delineate the molecular mechanism of PD-L1 expression to select the agent(s) likely to exhibit therapeutic activity for individual patient tumors.

Pancreatic ductal adenocarcinoma (PDAC) is a highly lethal human cancer with a total 5-year survival rate of only 7%.^4^ Approximately 80% of patients with pancreatic cancer lose the opportunity for surgical resection. Although there is marginal progress in chemotherapy, pancreatic cancer remains a malignancy of poor prognosis; therefore, novel therapeutic strategies are urgently needed for PDAC treatment. PD-L1 is expressed in PDACs, and its overexpression is associated with a poor prognosis.^5^ Preclinical investigations have shown that blocking PD-L1 inhibits pancreatic cancer development in animal models, suggesting that the PD-1/PD-L1 pathway could be a potential therapeutic target against PDAC.^6^ However, clinical trials have shown that targeting the PD-1/PD-L1 pathway with a single agent has only a modest effect in patients with PDAC,^7–10^ suggesting that in addition to PD-L1 expression, other targets for the treatment of PDAC must be identified.

Recently, we identified several intrinsic cell signaling pathways involved in reprogramming the immunosuppressive microenvironment of PDAC.^11–13^ We have recently reported that Forkhead box protein 3 (FOXP3), first defined as a key factor in regulatory T cells (Treg cells), is highly expressed in PDAC.^12^ In PDAC, it is referred to as cancer-FOXP3 (C-FOXP3), and its function is to mediate immune evasion by recruiting FOXP3+ T cells (Treg cells) via upregulation of CCL5. Interestingly, accumulating evidence has linked the coexistence of tumor-associated PD-L1 and Treg cell infiltration in multiple tumors, including PDAC,^14–16^ suggesting that C-FOXP3 may be correlated with PD-L1 expression in PDAC. The regulatory mechanism(s) of PD-L1 expression on pancreatic cancer cells remains poorly investigated. The inflammatory cytokine IFN-γ is known to trigger the upregulation of PD-L1, while recent literature suggests that oncogenic signals such as RAS mutation,^17^ MYC^18^, and MLL1^19^ also promote PD-L1 expression in cancer cells.

In the present study, we sought to determine whether C-FOXP3 induces PD-L1 expression in PDAC. If this were found to be the case, C-FOXP3 could represent a common driver controlling PD-L1 levels in PDAC cells and regulating the infiltration of Treg cells into PDAC tumors. In addition, based on our findings that blockade of CCL-5 achieved an effective response in patients with PDAC, we further evaluated the efficacy of combined blockade of PD-L1 and CCL-5 in these patients, especially those with high levels of C-FOXP3.

## Results

### PD-L1 correlates with C-FOXP3 expression in human PDAC

**Fig. 1:**
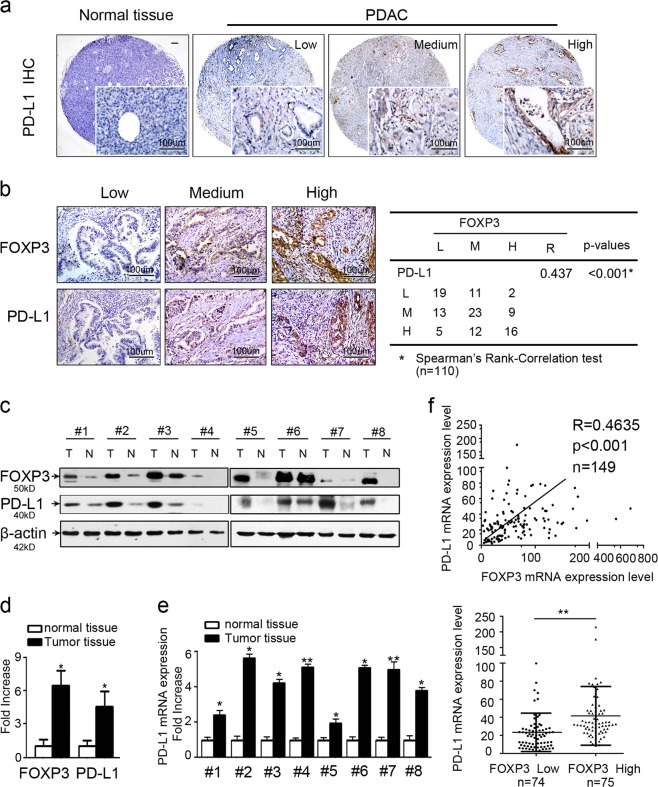
PD-L1 correlates with C-FOXP3 expression in human pancreatic ductal adenocarcinoma (PDAC). **a** Representative samples of PD-L1 staining in normal pancreatic tissues and PDAC tissues (brown) by immunohistochemistry. Magnification: ×40 and ×200. **b** (Left) PD-L1 and FOXP3 expression in PDAC tissues by immunohistochemistry (magnification: ×200). (Right) Statistical analysis of immunohistochemical results of PD-L1 and FOXP3 expression in 110 human PDAC samples. R (correlation coefficient) and *p* values were calculated by Spearman’s rank-correlation test. **c** Western blot analysis of PD-L1 and FOXP3 levels in eight total paired human PDAC tumors and matched adjacent normal tissues. PD-L1 and FOXP3 protein expression levels were normalized to those of β-actin (N: normal; T: tumor). **d** PD-L1 and FOXP3 protein expression levels in PDAC specimens versus paired adjacent normal tissues. Histogram (columns: mean, bars: standard deviation, *n* = 8). *p* values were calculated by Student’s *t*-test, *p < 0.05. **e** PD-L1 mRNA expression levels in PDAC specimens versus paired adjacent normal pancreatic tissues by RT-PCR analysis. Histogram (columns: mean, bars: standard deviation, *n* = 3). *p* values were calculated by Student’s *t*-test, *p < 0.05, **p < 0.01. **f** (Upper) Correlation between PD-L1 and FOXP3 mRNA expression in samples from 149 patients with PDAC from The Cancer Genome Atlas (TCGA). Spearman’s rank-order correlation test was applied to measure the strength of the association between PD-L1 and FOXP3 mRNA levels (*R* = 0.4635, *p* < 0.001). (Lower) PD-L1 expression in patients with FOXP3-low tumors (*n* = 74) and FOXP3-high tumors (*n* = 75); *p* values were calculated by the Mann–Whitney test, **p < 0.01

PD-L1 has been shown to be upregulated in PDAC, but the positive rates vary substantially due to differences in the antibodies used, the detection methods, and the values considered significant. To reconcile these known differences in the observed expression levels of PD-L1, three clonal antibodies were used to assess its expression in PDAC based on a standard immunohistochemistry protocol. There was good agreement between the three types of PD-L1 antibodies (Supplementary Fig. 1), indicating the stability of the detection protocol. The monoclonal antibody (Abcam 205921) containing PD-L1 clone 28-8 was used to define the PD-L1 levels for further analyses. We found that PD-L1 was expressed in the cytoplasm and on the membrane of PDAC cells. Furthermore, PD-L1 was expressed in some infiltrating lymphocytes as well as stromal cells. Among the 110 tumors evaluated in this study (from Tianjin Medical University Cancer Institute and Hospital, named the Tianjin cohort), 33 (~30.0%) had high PD-L1 expression levels (Fig. 1a). To further confirm the expression levels of PD-L1 in PDAC, we chose another cohort from the Affiliated Hospital of Qingdao University (99 tissues matched with the Tianjin cohort for age, sex, TNM stage, and differentiation, named the Qingdao cohort). As shown in Table 1, the two cohorts did not show a significant difference in the constituent ratio of PD-L1 expression (*p* = 0.709, calculated by the chi-square test).

**Table 1:** PD-L1 expression of two independent study cohorts by Immunohistochemistry

	Tianjin cohort	Qingdao cohort	*p*-values
*Gender*			
Male	58	59	0.318
Female	52	40	
Age^a^	58 (12)	63 (12)	0.438
*T stage*			0.679
1/2	53	51	
3	57	48	
*PD-L1*			0.709
Low	32	27	
Medium	45	37	
High	33	35	
*Differentiation*			0.663
Well	20	13	
Moderate	57	66	
Poor	33	20	
*pTNM stage*			0.152
IA/Ib	23	30	
IIA/IIb	87	69	

*p*-values were calculated by Chi-Square test

^a^*p*-values were calculated by Mann–Whitney U-test

Interestingly, the expression of PD-L1 and C-FOXP3 was colocalized in consecutive sections of PDAC tissues, and importantly, C-FOXP3 expression levels were correlated with high PD-L1 levels in the tumor cells (*R* = 0.437, *p* < 0.001) (Fig. 1b).

Next, we assessed the PD-L1 mRNA and protein levels in frozen PDAC tissues compared to paired normal tissues. Despite interindividual variations, PD-L1 protein (Fig. 1c, d) and mRNA (Fig. 1e) levels were elevated in PDAC tissues compared to adjacent normal tissues, indicating that PD-L1 was upregulated at the transcriptional level in PDAC.

We then investigated the correlation between FOXP3 expression and PD-L1 (CD274) in TCGA samples from 149 patients with PDAC obtained from the Cancer Genome Atlas website. First, we compared the mRNA levels of FOXP3 and PD-L1 and found a significantly positive correlation (*R* = 0.4635, *p* < 0.001) (Fig. 1f). A second analysis in which PD-L1 expression in PDAC tumors with low FOXP3 (*n* = 74) was compared with tumors exhibiting high FOXP3 (*n* = 75) revealed that high FOXP3 tumors had significantly higher PD-L1 levels than low FOXP3 tumors (*p* < 0.01) (Fig. 1f). In conclusion, PD-L1 is overexpressed in PDACs, and its expression correlates with C-FOXP3 expression at both the transcriptional and protein levels.

### C-FOXP3 directly activates PD-L1 transcription in murine and human PDAC cells

**Fig. 2:**
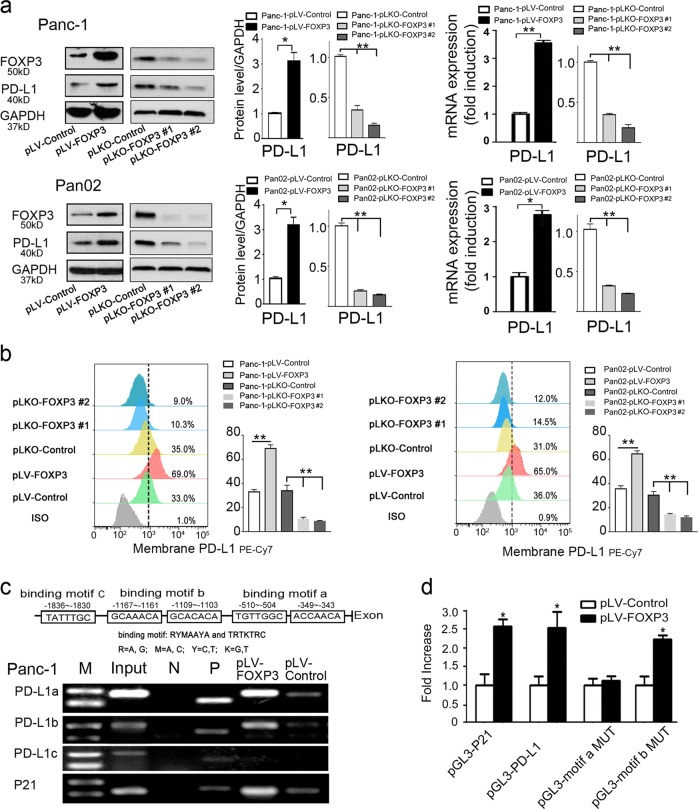
C-FOXP3 directly activates PD-L1 transcription in PDAC cells. **a** Establishment and verification of stable cell lines overexpressing or underexpressing FOXP3. pLV-control and pLV-FOXP3 indicate lentivirus vectors for the control and overexpression of C-FOXP3; pLKO-control and pLKO-FOXP3 indicate lentivirus vectors for the control and knockdown of C-FOXP3. (Left) Representative samples of C-FOXP3 and PD-L1 in Panc-1 and Pan02 cell lines detected by western blotting. GAPDH was used as an internal control. (Right) The protein and mRNA levels of PD-L1 were measured by western blot (GAPDH as a control) and real-time PCR (β-actin as a control). Histogram (columns: mean, bars: standard deviation, *n* = 3), *p* values were calculated by Student’s *t*-test, *p < 0.05, **p < 0.01. **b** Pancreatic cancer cell lines were stained with IgG isotype control and PD-L1-specific monoclonal antibody and analyzed for PD-L1 protein levels by flow cytometry. Quantification of PD-L1 protein levels on different pancreatic cancer cell surfaces. Columns: mean, bars: standard deviation, *n* = 3, *p* values were calculated by Student’s *t*-test, *p < 0.05, **p < 0.01. **c** (Upper) Specificity of the ChIP assay. The human PD-L1 gene, including the FOXP3 binding motif a to c. (Lower) Binding of FOXP3 and the PD-L1 promoter was observed at motifs a and b by PCR analysis. P21 was used as a positive control. N: negative control; P: positive control. **d** Panc-1 cells were transfected with either vector control or FOXP3 in conjunction with the luciferase reporter pGL3-PD-L1 (wild type), pGL3-motif a MUT or pGL3-motif b MUT (mutation of motif-a or motif-b) vectors. The pGL3-p21 promoter was used as a positive control. After 48 h, firefly and Renilla luciferase activities were measured using the Dual-Luciferase Reporter assay (Promega), and the ratio was determined. Histogram (columns: mean, bars: standard deviation, *n* = 3). *p* values were calculated by Student’s *t*-test, *p < 0.05

We further investigated whether C-FOXP3 regulated PD-L1 expression in human and mouse PDAC cell lines. Stable cell lines with either overexpressed or underexpressed C-FOXP3 were established. Overexpression of C-FOXP3 in human Panc-1 and mouse Pan02 cells correlated well with increased PD-L1 protein and mRNA levels. Moreover, knockdown of C-FOXP3 expression decreased PD-L1 protein and mRNA levels (Fig. 2a). In addition, the membrane expression of PD-L1 (determined by flow cytometry) was closely related to C-FOXP3 expression levels (Fig. 2b). Similar results were also obtained with MIA PaCa-2 and AsPC-1 cells (Supplementary Fig. 2a–c). Taken together, these results indicate that C-FOXP3 upregulates PD-L1 expression in PDAC tumor cells.

To determine the mechanism of C-FOXP3-induced transcription of PD-L1, we searched for potential FOXP3 binding sites in the proximal promoter region of the human PD-L1 gene and found three putative binding motifs (Fig. 2c). Chromatin immunoprecipitation (ChIP) assay was performed with FOXP3 antibody in Panc-1 cells, and the ChIP complexes were analyzed by PCR. As shown in Fig. 2c, FOXP3 directly bound to the PD-L1 promoter at motifs a and b, comparable to their binding to an established motif in P21. To determine whether motif-a and motif-b were the transcriptionally active sites, a series of luciferase reporter vectors containing either the wild-type PD-L1 promoter or the PD-L1 promoter with mutated binding motif-a or motif-b was constructed and transfected into Panc-1 cells with or without the C-FOXP3 expression plasmids.luciferase assay showed that overexpression of FOXP3 activated the luciferase reporter gene under the control of the PD-L1 promoter (Fig. 2d). However, mutation of the binding motif-a completely reversed the activity. Consistently, ChIP and luciferase assays showed that FOXP3 trans-activated PD-L1 in Pan02 murine PDAC cells (Supplementary Fig. 2d, e), suggesting that the regulatory role of FOXP3 in PD-L1 is a conserved mechanism. Taken together, we provide evidence that C-FOXP3 directly binds to motif-a of the PD-L1 promoter and activates its transcription in pancreatic cancer cells.

### Functional measure of C-FOXP3-induced upregulation of tumoral PD-L1

**Fig. 3:**
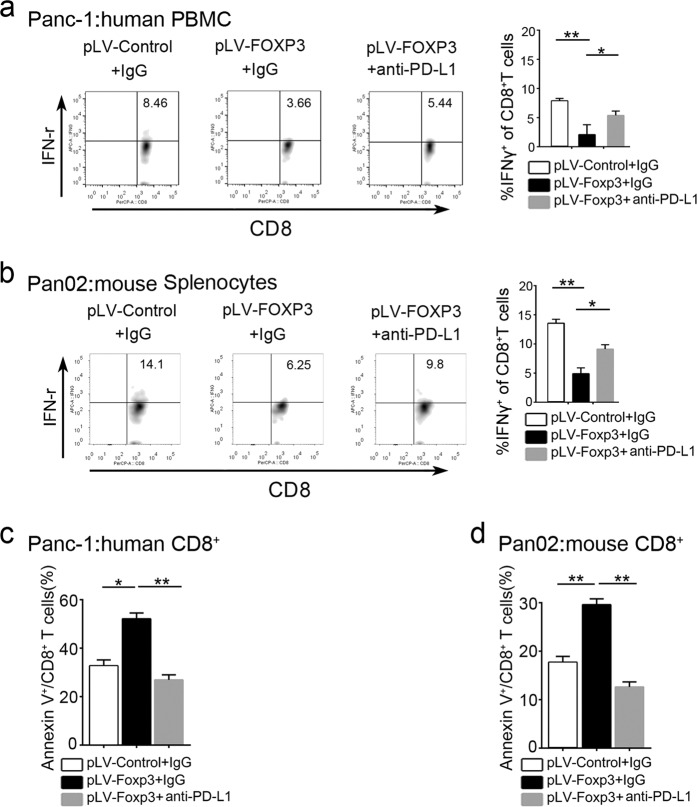
Functional measure of C-FOXP3-induced upregulation of tumoral PD-L1. **a** Human peripheral blood mononuclear cells (PBMCs) were isolated from healthy volunteer peripheral blood and stimulated with IL-2, anti-CD3 and anti-CD28 mAbs for 48 h. Then, PBMCs were cocultured with Panc-1-pLV-control or Panc-1-pLV-FOXP3 (pLV-control and pLV-FOXP3 indicate lentivirus vectors for control and overexpression of C-FOXP3) in the absence or presence of anti-PD-L1 for 18 h. The expression of IFNγ^+^ on CD8^+^ T cells was determined by flow cytometry. **b** Mouse splenocytes were isolated from the spleens of C57BL/6 mice and stimulated with IL-2, anti-CD3 and anti-CD28 mAbs for 48 h. Then, splenocytes were cocultured with Pan02-pLV-control or Pan02-pLV-FOXP3 in the absence or presence of anti-PD-L1 for 18 h. The expression of IFNγ^+^ on CD8^+^ T cells was determined by flow cytometry. **c** Human CD8^+^ T cells were isolated from healthy volunteer peripheral blood and stimulated with IL-2, anti-CD3, and anti-CD28 mAbs for 48 h. Then, CD8^+^ T cells were cocultured with Panc-1-pLV-control or Panc-1-pLV-FOXP3 in the absence or presence of anti-PD-L1 for 18 h. The expression of Annexin V^+^ CD8^+^ T cells was determined by flow cytometry. **d** Mouse CD8^+^ T cells were isolated from the spleens of C57BL/6 mice bearing Pan02-pLV-control tumors and stimulated with IL-2, anti-CD3 and anti-CD28 mAbs for 48 h. Then, CD8^+^ T cells were cocultured with Pan02-pLV-control or Pan02-pLV-FOXP3 in the absence or presence of anti-PD-L1 for 18 h. The expression of Annexin V^+^ CD8^+^ T cells was determined by flow cytometry. Histogram (columns: mean, bars: standard deviation, *n* = 3), *p* values were calculated by Student’s *t*-test, *p < 0.05, **p < 0.01

PD-L1 on tumor cells is known to inhibit the activity and toxic effects of CD8^+^ T cells.^20^ To measure the function of PD-L1 induced by C-FOXP3, peripheral blood mononuclear cells (PBMCs) or isolated CD8+ T cells (Supplementary Fig. 3) were stimulated by anti-CD3/anti-CD28 antibodies and cocultured with human or mouse PDAC cells. The activity of CD8^+^ T cells was determined by measuring IFN-γ secretion via flow cytometry. We showed that overexpression of FOXP3 in PDAC cells significantly inhibited IFN-γ production in CD8^+^ T cells. Treatment with anti-PD-L1 antibody partially restored the IFN-γ levels from CD8^+^ T cells cocultured with human or murine pLV-FOXP3 PDAC cells (Fig. 3a, b and Supplementary Fig. 4a). In addition, we measured the apoptosis of CD8^+^ T cells cocultured with human or murine PDAC cells. When cocultured with PDAC cells, CD8^+^ T cells showed an increased apoptotic index in the C-FOXP3 overexpression group, which was reversed by the PD-L1 antibody (Fig. 3c, d Supplementary Fig. 4b). In contrast, we measured IFN-γ production in CD8^+^ T cells cocultured with human or murine pLKO-FOXP3 (FOXP3 knockdown) PDAC cell lines by the same coculture system. We found that pLKO-FOXP3 exhibited higher IFN-γ production relative to the pLKO-control groups. Furthermore, the apoptotic index of CD8^+^ T cells decreased substantially in the pLKO-FOXP3 group (Supplementary Fig. 4c–f). These data suggest that C-FOXP3 induces the functional expression of immunosuppressive PD-L1 on PDAC cells.

### Anti-PD-L1 antibody counteracts the growth of PDAC induced by C-FOXP3 in vivo

**Fig. 4:**
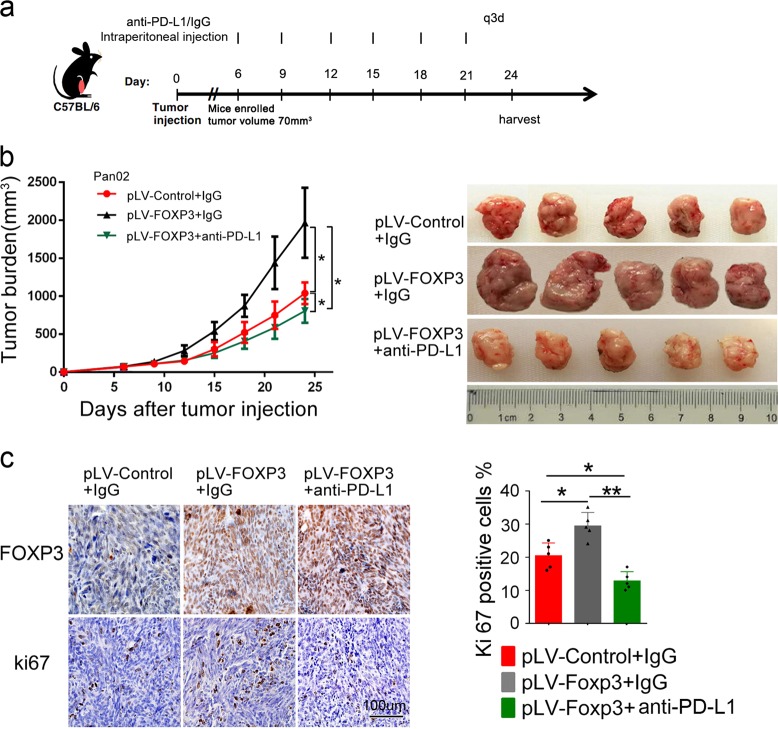
Anti-PD-L1 antibody counteracts the growth of PDAC induced by C-FOXP3 in vivo. **a** C57BL/6 mice were inoculated subcutaneously with murine pancreatic tumor cells in their right flanks (5 mice/group). When tumors reached approximately 70 mm^3^ in size, mice were treated with isotype control or anti-PD-L1 for 3 weeks. **b** Mean (left) and individual (right) tumor volumes over time. On day 24, tumor growth was evaluated by measuring tumor volumes and compared statistically by one-way ANOVA with the Bonferroni post hoc test. (*p < 0.05). Line chart: points: mean, bars: standard deviation, *n* = 5. **c** (Left) Immunohistochemical staining of FOXP3 and Ki67 in tumor slices. Representative images are shown, magnification: ×200. Ki67 (right) expression levels for the slides are summarized in the graph. Histogram (columns: mean, bars: standard deviation, *n* = 5), *p* values were calculated by one-way ANOVA tests, *p < 0.05, **p < 0.01

To further evaluate the potential antitumor effect of PD-L1 antibody in vivo, mice inoculated with Pan02-pLV-FOXP3 tumor cells were treated with anti-PD-L1 antibody or control IgG (200 μg, intraperitoneal injection q3d) for 3 weeks (Fig. 4a) when the tumor volume reached approximately 70 mm^3^. Tumor growth was assessed during and after treatment (Fig. 4b). Consistent with the results of our previous study,^12^ C-FOXP3 promoted tumor growth in immunocompetent mouse models. However, PD-L1 blockade partially reversed this growth (Fig. 4c). In addition, Ki67 levels in the tumor sections demonstrated that anti-PD-L1 suppressed the proliferation of PDAC cells in the models (Fig. 4d). We further found that anti-PD-L1 did not affect the proliferation and apoptosis of pancreatic cancer cells (Supplementary Fig. 5a, b) in vitro, suggesting that the antitumor effect of PD-L1 blockade is mediated via revoking the immune attack. Collectively, these data suggest that the PD-1/PD-L1 pathway mediates the proliferation of PDAC induced by C-FOXP3.

### PD-L1 expression correlates with the accumulation of FOXP3^+^ Treg cells in PDAC

**Fig. 5:**
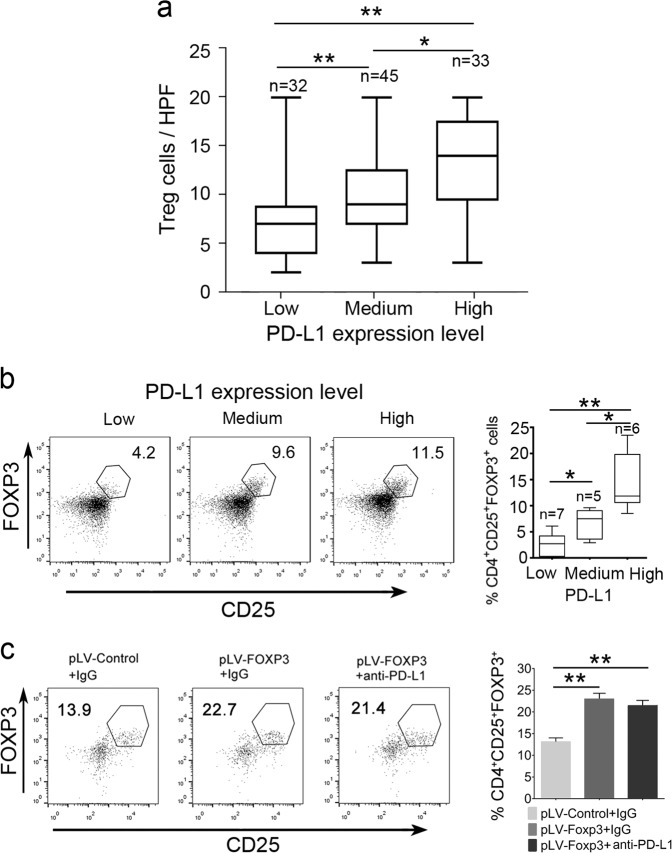
PD-L1 expression is correlated with the accumulation of FOXP3^+^ Treg cells in PDAC. **a** Statistical analysis of FOXP3^+^ Treg cell accumulation in the PDAC microenvironment with low, medium and high expression of PD-L1 (*n* = 110). Box plot presentation (the median horizontal line represents the median value, and the upper and lower lines represent the 25th and 75th percentiles, respectively), *p < 0.05, **p < 0.01 by the Mann–Whitney test. **b** (Left) Flow cytometry analysis (FACS) of FOXP3^+^ Treg cell accumulation in tumor tissues from fresh surgical samples (*n* = 18). (Right) Summary data are shown as box plot presentation (the median horizontal line represents the median value, and the upper and lower lines represent the 25th and 75th percentiles, respectively), *p < 0.05, **p < 0.01 by the Mann–Whitney test. **c** Treatment of PDAC xenografts with anti-PD-L1 antibody. (Left) Tregs were counted by FACS. pLV-control and pLV-FOXP3 indicate lentivirus vectors for the control and overexpression of C-FOXP3. (Right) Histogram (columns: mean, bars: standard deviation, *n* = 5), *p < 0.05, **p < 0.01. *p* values were calculated by one-way ANOVA tests

We have recently reported that C-FOXP3 promotes Treg cell infiltration in PDAC.^12^ Based on our present findings that C-FOXP3 upregulates the expression of tumoral PD-L1, the number of Treg cells should be correlated with tumoral PD-L1 levels. Consistent with this deduction, immunochemical analysis indicated that the number of Treg cells paralleled the tumoral PD-L1 expression in PDAC samples (Fig. 5a). To further confirm this finding, flow cytometry was performed on 18 fresh PDAC tissues. We demonstrated that PDAC with higher PD-L1 levels showed higher infiltration of Treg cells (Fig. 5b). Further analysis showed that treatment of PDAC xenografts with anti-PD-L1 antibody had no effect on the proportion of infiltrating Treg cells (Fig. 5c), suggesting that C-FOXP3 regulates the number of Treg cells through a PD-L1-independent mechanism.

### Anti-PD-L1 antibody enhances the antitumor effect of CCL-5 blockade in PDAC in mice with high C-FOXP3 levels

**Fig. 6:**
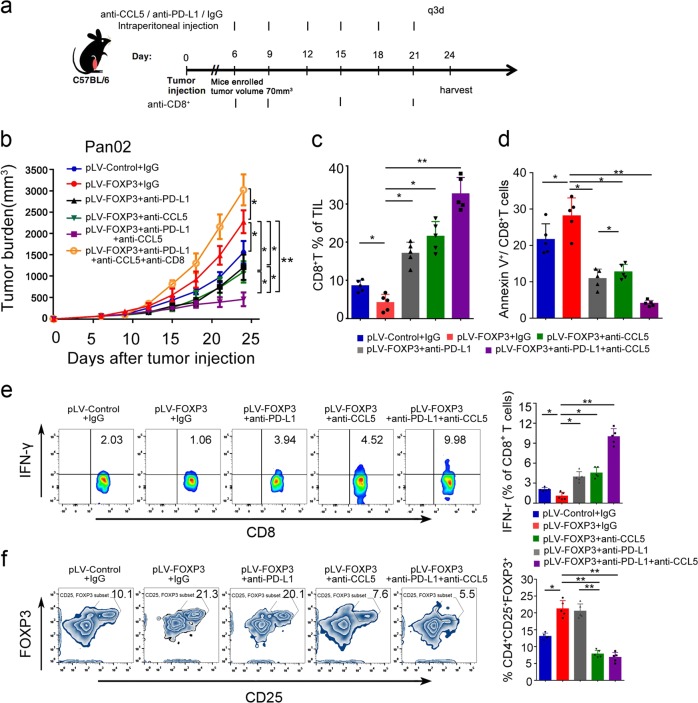
Anti-PD-L1 antibody enhances the antitumor effect of CCL5 blockade in PDAC in mice with high C-FOXP3 levels. **a** C57BL/6 mice were inoculated subcutaneously with Pan02-pLV-control or Pan02-pLV-FOXP3 murine pancreatic tumor cells in their right thoracic flanks. When tumors reached approximately 70 mm^3^, mice were treated with 200 μg (intraperitoneal injection q3d) of isotype control. pLV-control and pLV-FOXP3 indicate lentivirus vectors for control and overexpression of C-FOXP3. **b** Tumor growth was evaluated by measuring tumor volumes and compared statistically by one-way ANOVA with the Bonferroni post hoc test. Line chart, points: mean, bars: standard deviation. *n* = 5, *p < 0.05, **p < 0.01. **c**, **d** Flow cytometry analysis of CD8^+^ T cell percentages of total tumor infiltrating lymphocytes (TILs) in the tumor microenvironment between the control IgG and anti-CD8 injection groups. Summary data of flow cytometry analysis of apoptotic CD8^+^ T cells in the tumor microenvironment between different groups are shown as histograms (columns: mean; bars: standard deviation, *n* = 5, *p* values were calculated by one-way ANOVA with Bonferroni post hoc test, *p < 0.05, **p < 0.01. **e** (Left) Quantification of IFNγ-producing CD8^+^ T cells as percentages of total cells. IFNγ-producing CD8^+^ T cells were evaluated by FACS. (right) Histogram (columns: mean; bars: standard deviation, *n* = 5). *p* values were calculated by one-way ANOVA with Bonferroni post hoc test. *p < 0.05, **p < 0.01. **f** (Left) FACS analysis of Treg recruitment into the tumor microenvironment. (Right) Histogram (columns: mean; bars: standard deviation, *n* = 5). *p* values were calculated by one-way ANOVA with Bonferroni post hoc test. *p < 0.05, **p < 0.01

We have shown that C-FOXP3 promotes Treg cell infiltration by inducing CCL-5 secretion in PDAC. Moreover, blockade of Treg cell infiltration by CCL-5 antibody inhibits the growth of PDAC in mouse models exhibiting high C-FOXP3 levels.^12^ Here, we investigated the possibility that anti-PD-L1 antibody might synergize with anti-CCL5 antibody to augment the antitumor immune response. Mice inoculated with Pan02-pLV-FOXP3 cells were treated with PD-L1 and/or CCL-5 blocking antibodies (200 μg, intraperitoneal injection q3d) for 3 weeks, and when the tumor volumes reached approximately 70 mm^3^ (Fig. 6a), the effects of single or combined treatment on tumor growth were evaluated. Although CCL5 and PD-L1 antibodies alone reduced the tumor burden, the antitumor effect was more dramatic in the mice treated with both antibodies (Fig. 6b and Supplementary Fig. 6a, b).

We further investigated the immunomodulatory effect of the treatments. Tumor tissues were collected after 24 days of treatment with anti-CCL5, anti-PD-L1, or anti-CCL-5 plus anti-PD-L1. The combination of anti-CCL5 and anti-PD-L1 antibodies significantly upregulated the ratio of tumor-infiltrating CD8^+^ T cells (Fig. 6c). Additionally, the combined blockade suppressed the apoptotic rate of CD8^+^ T cells (Fig. 6d). In addition, tumor-infiltrating CD8^+^ T cells of the mice treated with anti-PD-L1 and/or anti-CCL5 antibodies exhibited enhanced IFN-γ production relative to the control isotype mice, especially in the combination treatment group (Fig. 6e). In accordance with our previous findings, treatment with anti-CCL5 antibody reduced the infiltration of Treg cells in tumor tissues (Fig. 6f), but the addition of PD-L1 antibody did not augment the effect. To investigate whether the effect of the combined blockade was CD8^+^ T cell dependent, Pan02-pLV-FOXP3-bearing mice were administered CD8^+^ depleting antibodies during the process of treatment. Depletion of CD8^+^ T cells completely counteracted the effects of tumor inhibition produced by the combination of anti-PD-L1 and anti-CCL5 antibodies, indicating that CD8^+^ T cells are critical for the antitumor effects of the combination treatment (Fig. 6b and Supplementary Figs. 3c, 6a, b).

**Fig. 7:**
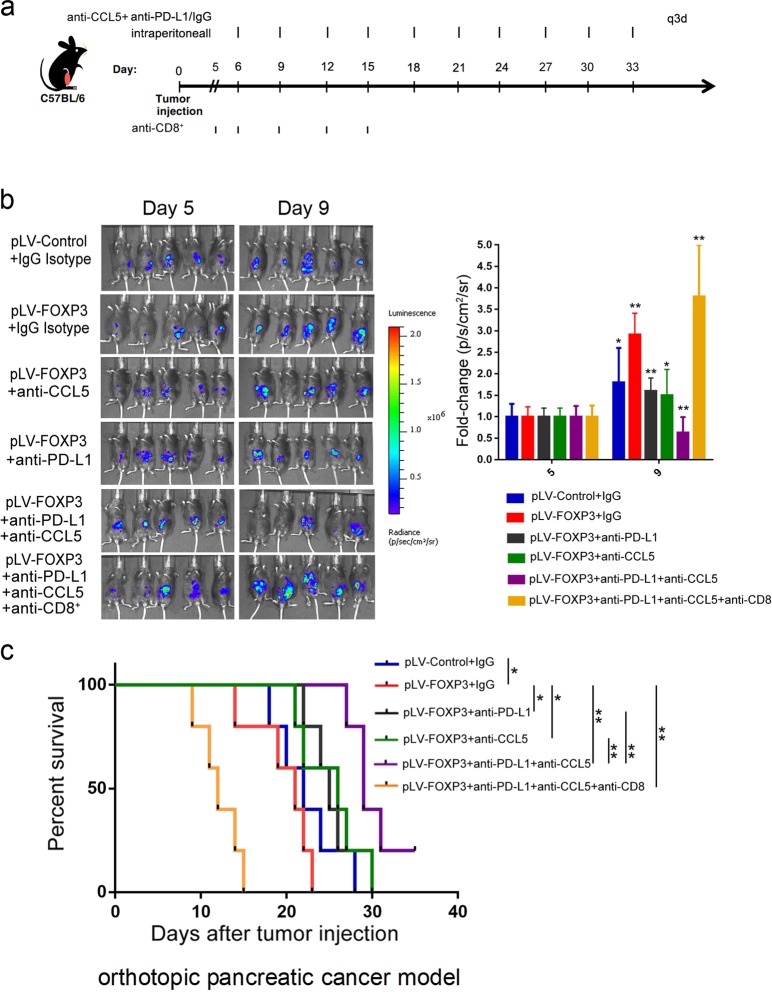
CCL-5 and PD-L1 antibody combined blockade increases overall survival of the orthotopic model of pancreatic cancer. **a** C57BL/6 mice were orthotopically injected with Pan02-pLV-control-Luc (lentivirus vectors with luciferase gene stably expressed in Pan02 cells) or Pan02-pLV-FOXP3-Luc (lentivirus vectors with FOXP3 and luciferase gene stably expressed in Pan02 cells). Mice were randomized and treated with anti-CCL5, anti-PD-L1, anti-CCL5 + anti-PD-L1, anti-CCL5 + anti-PD-L1 + anti-CD8 or isotype control Abs until the study endpoint. **b** (left) Tumor growth was assessed by bioluminescent imaging. (right) Quantification of the bioluminescent imaging signals on day 5 and day 9. The results are expressed as the fold change relative to those on day 5. Histogram (columns: mean; bars: standard deviation, *n* = 5), *p* values were calculated by paired *T*-test. *p < 0.05, **p < 0.01. **c** Kaplan–Meier survival curves with log-rank test for significance between different groups (*p < 0.05, **p < 0.01)

We further examined the survival time of combination therapies in an orthotopic model of PDAC. Pan02-pLV-control-luc or Pan02-pLV-FOXP3-luc cells were orthotopically injected into the pancreatic tissues of C57BL/6 mice. Tumor growth was assessed by bioluminescent imaging. When tumors were detectable by imaging, the models were randomized and treated with anti-CCL5, anti-PD-L1, anti-CCL5 + anti-PD-L1, anti-CCL5 + anti-PD-L1 + anti-CD8 or isotype control antibodies until the study endpoint (Fig. 7a). Quantification of the bioluminescent imaging signal over time demonstrated a progressive increase in tumor growth. We observed a decreased tumor burden in mice with combined treatment compared to the isotype control, anti-CCL5, or anti-PD-L1 alone (Fig. 7b). In addition, treatment with combined anti-CCL5 and anti-PD-L1 antibodies significantly improved overall survival (OS) compared to the isotype control, anti-CCL5 or anti-PD-L1 alone (Fig. 7c).

## Discussion

In this report, we have shown that C-FOXP3 upregulates PD-L1 levels in human and mouse PDAC cells by binding directly to motif-a of the PD-L1 promoter. Further functional studies have indicated that tumoral PD-L1 inhibits CD8^+^ T cell survival and activity induced by C-FOXP3. We and others have shown that C-FOXP3 serves as an oncogene to predict poor prognosis and remodel the immune microenvironment by recruiting Treg cells^12^ and inhibiting CD4^+^ Th cells.^21^ The present findings extend the function of C-FOXP3 by showing that it directly inhibits the activity of CD8^+^ T cells via the PD-L1/PD-1 pathway. Thus, C-FOXP3 represents a core transcription factor that mediates the immune escape of PDAC. Due to the complexity of the immune microenvironment of PDAC, previous studies used multiplex parameters to stratify the immunological subtypes of PDAC based on the availability of sufficient tissue samples from surgical resection.^22–24^ However, 80–85% of patients with PDAC are unresectable at diagnosis, and only limited biopsy samples are available for pathological analysis. Thus, an ideal biomarker for detecting an immunosuppressive microenvironment is a cell-intrinsic protein that can be used for selecting patients likely to have a good response to immunotherapy based upon analysis of biopsy samples. The present study suggests that c-FOXP3 could be a candidate cell-intrinsic biomarker for personalizing PDAC immunotherapy.

The prevalence of checkpoint blockade immunotherapy inspired multiple studies focusing on the expression and regulatory mechanisms of PD-L1 in tumor cells. With respect to pancreatic cancer, previous studies reported divergent tumoral PD-L1 levels, ranging from 12 to 90%.^5,25,26^ This variance in PD-L1 levels may result from the specificity of the detection methods, the quality of the samples and the quality and sensitivity of the detection antibody. To cautiously define the expression of PD-L1, we optimized the immunohistochemical protocols with three different PD-L1 antibodies and selected samples from patients without neoadjuvant chemotherapy. Finally, tumoral PD-L1 expression was evaluated by two experienced pathologists working independently. We observed that approximately 30% of PDAC cells highly expressed PD-L1, which is consistent with the data from a recent meta-analysis.^27^

Although tumoral PD-L1 can function as a marker in NSCLC,^28,29^ antibodies to PD-L1 alone should not be introduced as single-agent therapy for PDAC because only a few patients with mismatch repair deficiency (approximately 3%) respond to the treatment. Accumulating evidence suggests that a few molecules combined with anti-PD-1/PD-L1 showed enhanced treatment potential in PDAC, such as CXCL12, CD40, and IL-6. For example, targeting CXCL12 could enhance the tumor growth-suppressing effect of PD-1/PD-L1.^30^ In addition, combined blockade of IL-6 and PD-1/PD-L1 could enhance the effective T cell infiltration into tumors.^31^ We recently showed that CCL-5 was upregulated by C-FOXP3 to recruit Treg cells into PDAC.^12^ Thus, PD-L1 could be a promising strategy to enhance the effect of CCL-5 antibody to treat PDAC, especially PDACs with high C-FOXP3 levels. To investigate this possibility, we constructed an immunocompetent PDAC murine model bearing C-FOXP3^high^ Pan02 (murine pancreatic cancer cells) cell xenografts to evaluate responses to immunotherapy. In this model, the combined blockade of CCL-5 and PD-L1 synergistically suppressed tumor growth by stimulating the activity of CD8^+^ cytotoxic T cells. To further mimic the pancreatic microenvironment, the combination of CCL-5 and PD-L1 antibodies was used in an orthotopic murine model, which led to enhanced survival time of the mice, suggesting the potential translational value of this strategy.

It should be mentioned that the function of c-FOXP3 is complex and cell-type dependent. For instance, in colorectal cancer, breast cancer and gastric cancer, C-FOXP3 is a tumor suppressor gene, whereas in NSCLC, esophageal cancer and pancreatic cancer, it is an oncogene contributing to a poor prognosis from the tumor.^32^ Therefore, whether the regulatory role of C-FOXP3 on PD-L1 is a ubiquitous mechanism needs to be further determined.

In conclusion, we have demonstrated that PD-L1 is a direct target of C-FOXP3 in PDAC cells. Combined blockade of PD-L1 and CCL-5 may provide an effective therapy for patients with PDAC, especially those patients whose tumors have high C-FOXP3 levels.

## Materials and methods

### Patients and samples

PDAC samples used in this study were from patients given radical resection at Tianjin Medical University Cancer Institute and Hospital (*n* = 110) and The Affiliated Hospital of Qingdao University (*n* = 99). All patients received gemcitabine-based adjuvant chemotherapy for at least six cycles. Normal pancreatic tissues were selected from patients who underwent surgery for other diseases. The use of these tissues and patient information was authorized by the local ethics committee. All patients signed informed consent before their specimens and information were selected in accordance with the rules of the ethics committee. We did not involve patients or the public in our work.

### Quantitative real-time PCR

Total RNA samples were extracted with TRIZOL Reagent (Invitrogen). The SYBR Green-Based Real-Time Quantitative Reverse Transcription PCR system (TaKaRa, Dalian, China) was used to detect the mRNA levels. Primers are shown in Supplementary Table 1.

### Western blot analysis

The tumor tissues and PDAC cell lines were lysed with SDS lysis buffer supplemented with proteinase inhibitors (Sigma, Deisenhofen, Germany). The concentrations of protein were further measured by the BCA Protein Assay reagent (Pierce, Rockford, IL, USA). The antibodies used are shown in Supplementary Table 2.

### Immunohistochemistry

Paraffin-embedded PDAC specimens were cut and placed on slides. Immunohistochemical staining was performed using different antibody clones for PD-L1 from Abcam (28-8), Cell Signaling (E1L3N) and R&D (AF156). Further information is shown in the Supplementary Materials and Methods. Antibodies for immunohistochemistry are listed in Supplementary Table 2. Immunohistochemistry was performed by a standard protocol as described.^8^

### Quantification of Treg cells

Immunohistochemical detection of FOXP3^+^ T cells was performed in accordance with a previous protocol.^8^ Stained Treg cells were calculated using light microscopy. We then randomly chose 10 high-power fields (40× magnification) per specimen in a zone of 0.0625 mm^2^ for the digital photo. Two investigators, who were blinded to the histopathological characteristics of the specimens and patient outcomes, independently counted the Treg cells as well as the total lymphoid infiltrates.

### Data analysis of TCGA

TCGA data obtained from 149 patients with pancreatic carcinoma were downloaded from The Cancer Genome Atlas website: (https://www.cancer.gov/about-nci/organization/ccg/research/structural-genomics/tcga). Normalized counts of the mRNA expression of FOXP3 and PD-L1 were obtained and analyzed.

### Chromatin immunoprecipitation (ChIP) and luciferase analysis

To identify the role of FOXP3 in the immune regulation of pancreatic cancer, ChIP assays and luciferase assays were performed using a commercial kit following the manufacturer’s protocols. PCR primers are shown in Supplementary Table 1. Further information is provided in the Supplementary Materials and Methods.

### Flow cytometry analysis

The levels of PD-L1 on PDAC cells were determined by flow cytometry. Human and mouse tumor-infiltrating Treg cells, CD8^+^ T cell phenotypes and apoptotic CD8^+^ T cells were identified by flow cytometry. Cells were counted on the LSRFortessa flow cytometer (BD), and data were analyzed by FlowJo software (Tree Star, OR, USA). Details are shown in the Supplementary Materials and Methods.

### Human peripheral blood mononuclear cells (PBMCs), mouse splenocytes, CD8^+^ T cell isolation and coculture system

We constructed an in vitro coculture model to model the tumor microenvironment. Human PBMCs were isolated from healthy donors. The spleens of C57BL/6 mice were isolated to obtain splenocytes. The isolated cells were stimulated with recombinant interleukin 2 (rIL-2) (Sigma-Aldrich, St. Louis, MO, USA) and human T-activator CD3/CD28 (Gibco, Camarillo, CA, USA) for 48 h before coculture with the above-described tumor cell lines. Human CD8^+^ T cells were isolated from PBMCs by the CD8^+^ T cell isolation reagent (Miltenyi Biotec, Bergisch Gladbach, Germany. #130-096-495). For mouse CD8^+^ T cell isolation, fresh spleens from Pan02-pLV-control orthotopic tumor-bearing C57BL/6 mice were ground into small pieces, transferred to cell strainers (BD Biosciences, Bedford, MA, USA) and mechanically minced by a plunger. Cells were collected after passing the cell strainer and separated by Ficoll-Paque gradient centrifugation. Then, a CD8^+^ T cell isolation kit was used for isolation of mouse CD8^+^ T cells. Both human and mouse CD8^+^ T cells were stimulated with rIL-2 (30 U/mL) and T-activator CD3/CD28 at an equal ratio of bead to cell for 48 h before coculture with human or mouse pancreatic cancer cells.

Human PBMCs, mouse splenocytes or CD8^+^ T cells were plated in 96-well U-bottom plates with human or murine PDAC cell lines in 100 µL of RPMI-1640 complete media (cancer cell: hPBMC/splenocytes/CD8^+^ T cell = 1:5) in the presence of 100 mg/mL anti–PD-L1 antibody (anti-human PD-L1, Clone 29E.2A3 or anti-mouse PD-L1, Clone 10F.9G2; BioXcell) or isotype control (Clone MPC-11 or LTF-2, BioXcell). After 48 h of incubation, cells in the supernatants were marked and detected by flow cytometry. The numbers of human and mouse IFN-γ-producing CD8^+^ T cells and apoptotic CD8^+^ T cells were then quantified by flow cytometry analysis.

### Animals and tumor models

Mice were housed in specific pathogen-free (SPF) conditions, and animal protocols were authorized by the local ethics committee. For both the subcutaneous and orthotopic tumor models, mice were treated with isotype IgG (200 μg/mouse, q3d, clone LTF-2, BioXcell), anti-PD-L1 (200 μg/mouse, q3d, clone 10F.9G2, BioXcell) or combined with anti-CCL5 (20 μg/mouse, R&D, Minneapolis, MN) intraperitoneally q3d. The anti-CD8 antibody (No. 2.43, BioXCell, UK) for eradicating CD8^+^ T cells was *i.p*. injected at a dose of 250 µg on days 6, 9, 15, and 21. Tumor volumes were measured every three days with a caliper. For the orthotopic model, C57BL/6 mice were injected with 1 × 10^6^ Pan02-pLV-control-luc or Pan02-pLV-FOXP3-luc cells in Matrigel (BD Biosciences) into the pancreatic tail. Tumor growth was analyzed by bioluminescent imaging. The survival time of each mouse was recorded. Further information is provided in the Supplementary Materials and Methods.

### Statistical analyses

SPSS (21.0 version) was used for the statistical analysis. All experiments were independently performed three times, and the values are presented as the mean ± S.D. Differences between variables with normal distribution were determined by Student’s *t*-test or one-way ANOVA with post hoc analysis. The nonparametric statistical method of the Mann–Whitney *U*-test was introduced for variables with skewed distributions and unknown distributions, and values are presented as medians [interquartile ranges]. The Kaplan–Meier method was introduced for evaluating the median survival, and the difference between survival rates was examined by the log-rank test. The relationship between rank variables was analyzed by Spearman’s rank correlation test. *p* < 0.05 was considered significant.

## Supplementary information

supplemental materials

## Data Availability

The datasets for the current study can be obtained from the corresponding authors. The collected data are stored in anonymous form.
