# Supplementary material for: PD-L1 is a direct target of cancer-FOXP3 in pancreatic ductal adenocarcinoma (PDAC), and combined immunotherapy with antibodies against PD-L1 and CCL5 is effective in the treatment of PDAC
Source: Signal Transduct Target Ther. 2020 Apr 17;5:38. doi: 10.1038/s41392-020-0144-8 (PMC7162990; doi:10.1038/s41392-020-0144-8)
Supplement: Supplementary file 1 — supplemental materials [file 41392_2020_144_MOESM1_ESM.docx]

Supplementary Materials for

**PD-L1 is a Direct Target of Cancer-FOXP3 in Pancreatic Ductal Adenocarcinoma (PDAC) and Combined Immunotherapy with Antibodies Against PD-L1 and CCL5 is effective in the treatment of PDAC**

Xiuchao Wang, Xin Li, Xunbin Wei,Haiping Jiang, Chungen Lan, Shengyu Yang, Han Wang, Yanhui Yang, Jihui Hao, He Ren

Correspondence to: herenrh@163.com

**This PDF file includes:**

Materials and Methods

Supplemental Figure legends

Figures. S1 to S6

Tables S1 to S2

**Materials and Methods**

**Cell culture and transfection**

Human PDAC cell lines, Panc-1, AsPC-1 and MIA PaCa-2, were obtained from the Type Culture Collection Committee of the Chinese Academy of Sciences (Shanghai, China). For tumor immunology research, the murine PDAC cell line, Pan02, was a gift from Prof. Yang SY (Penn State College of Medicine, Hershey, PA, USA). Recently, all cell lines were authenticated by short-tandem repeat analysis using the Promega PowerPlex®1.2 analysis system (Genewiz Inc., Beijing). All cell lines were tested for the absence of mycoplasma by National Stem Cell Engineering Research Center (Tianjin, China). Based on the basal level of FOXP3, the stable cell lines for FOXP3 overexpression and knockout were constructed. All of these stable cell lines have been used in our previous studies [8]. All cell lines were grown at 37 °C in a humidified atmosphere of 95% air and 5% CO_2_ using either Dulbecco’s modified Eagle medium (DMEM) or RPMI-1640 medium with 10% fetal bovine serum (FBS).

**Immunohistochemistry**

The slides were deparaffinized in xylene and rehydrated through graded ethanol to water before staining. All sections were treated with EDTA (pH 8.0) for antigen retrieval and with 3% H_2_O_2_ for the inactivation of endogenous peroxidase. Immunohistochemical (IHC) stains were evaluated independently by two pathologists who were blinded to the clinical data. For c-FOXP3 staining, specific nuclear staining of epithelial tumor cells observed under a light microscope was considered positive. The score was determined using the following criteria: 0, negative; 1, low; 2, medium; 3, high. The extent of staining was scored as 0, 0% stained; 1, 1% to 25% stained; 2, 26% to 50% stained; 3, 51% to 100% stained. Five random fields (100×magnification) were evaluated under a light microscope. The final scores were calculated by multiplying the scores of the intensity with those of the extent and dividing the samples into four grades: < 3, low staining (+); 3 to 6, medium staining (++); >6, high staining (+++). For PD-L1 staining, the Tumor Proportion Score (TPS) is the percentage of viable tumor cells showing partial or complete membrane staining (≥1+) relative to all viable tumor cells present in the sample (positive and negative). Five random fields (100×magnification) per tumor were evaluated. A cut-off for PD-L1 positive stained tumor in each case was 1% of cells, the expression<1% was evaluated as negative. The immune cells were not evaluated. The final scores as low (L) with expression of ≤5% stained; medium (M), 6% to 9% stained; high (H) with expression of ≥10% stained.

**Plasmid construction and stable cell lines establishment**

Human and mice fused FOXP3 genes were amplified by PCR, using commercial FOXP3-overexpressed plasmids (InvivoGen) as the templates. Then, fused FOXP3 genes were cloned into pLV-EF1-MCS-IRES-Bsd vectors (Biosettia). Lentiviruses were produced in 293T cells for stable transfection of cell lines following the manufacturer’s instructions. The lentivirus vectors over-expressing reporter gene of GFP (pLV-GFP) was used for control of the transfection efficiency and viral titer. Empty vector was transfected into the same cell lines as control. A total of 1 × 10^5^ tumor cells in 2 ml medium with 8μg/ml polybrene were infected with 1 ml lentivirus supernatant. 48hrs later, blasticidin (InvivoGen) was added for selection. For the stable knockdown cell lines, shRNA sequences were designed by sigma shRNA designer (http://sigmaaldrich.com/). Three recommended sequences for FOXP3 genes were synthesized and cloned into the pLKO.1-puro-Vectors. Lentiviruses were produced in 293T cells. Scramble sequences were transfected into the same cell line to use as control. Among the three stable cell lines, the most efficient one was used for relevant assays.

**Quantitative real-time PCR**

Relative quantities of mRNA expression were analyzed using real-time PCR (ABI Prism 7500 Sequence Detection System, Applied Biosystems). The SYBR green fluorescence dye was applied in this experiment.

**Western Blot**

Protein lysates (30 μg) were separated by SDS-PAGE, and target proteins were detected by Western blot analysis using antibodies against PD-L1 (1:1000), FOXP3 (1:1000) and β-actin (1:5000) (Supplementary Table **2**). Secondary antibodies: Goat anti-rabbit or mouse antibody at 1:5000 dilutions (Abmart). Specific proteins were visualized using an enhanced chemiluminescence detection reagent (Pierce).

**Chromatin immunoprecipitation (Ch-IP) and luciferase analysis**

Chromatin immunoprecipitation assays were performed using a commercial kit according to the manufacturer’s instructions (Millipore, Burlington, MA, USA). PCR primers are listed in Supplemental Table **1**. Luciferase analysis was performed according to the binding sites identified in Ch-IP analysis using wild-type and mutated promoters of PD-L1 and P21. Genomic DNA fragments of the human and mice PD-L1 gene, the transcription initiation site was generated by PCR and inserted into pGL3-Basic vectors (nominated pGL3-PD-L1). All constructs were sequenced to confirm their identity. Luciferase activity was measured using the Dual-Luciferase Reporter Assay System (Promega, Fitchburg**,** WI, USA).

**Antibodies and flow cytometry analysis**

The following monoclonal antibodies (mAbs) purchased from Biolegend (San Diego, CA, USA) and eBiosciences (San Diego, CA,USA) were used for staining: APC anti-human CD274 (B7-H1, PD-L1) antibody, isotype control APC mouse IgG2b, κ isotype Ctrl; APC anti-mouse CD274 (B7-H1, PD-L1) antibody, isotype control APC rat IgG2b, κ Isotype Ctrl; FITC anti-human CD8a (HIT8a), APC anti-human IFN-γ (4S.B3), Human Treg Flow Kit (FOXP3 Alexa Fluor® 488/CD25 PE/CD4 PerCP) (BioLegend #320127), FITC anti-mouse CD8a(53-6.7), APC anti-mouse IFN-γ (XMG1.2) , Annexin V-APC/PI (KeyGEN BioTECH # KGA1030-100). After routine culture, FOXP3 stabilized pancreatic cancer cell lines were harvested and washed with cold PBS, then stained with human or mouse anti-PD-L1. Tumor tissues were digested into single-cell suspensions using the digestion buffer containing 1 mg/ml collagenase, 2.5 U/ml hyaluronidase, and 0.1 mg/ml DNase. All cells were washed with cold PBS. Cells were then filtered through 70-mm cell strainers and single-cell suspensions stained with the antibodies. Human and mouse tumor infiltration Treg cells were identified by flow cytometry. Then, human and mouse tumor infiltration Treg cells, CD8^+^ T cells, CD8^+^ T cell phenotype and apoptotic CD8^+^ T cells were analysed. Isotype controls were used as negative controls. Cells were analyzed on an LSRFortessa (BD) flow cytometer, and data analyzed using FlowJo (Tree Star, OR, USA).

**Animals and tumor models**

All mice were maintained in specific pathogen-free conditions and animal protocols were approved by the Ethics Committee of Tianjin Medical University Cancer Institute and Hospital, in compliance with the principles and procedures of the NIH Guide for the Care and Use of Laboratory Animals. Four to six-week old female C57BL/6 mice were used. After the first week of quarantine, a total of 1×10^6^ tumor cells were subcutaneously or orthotopically injected into each mouse to form tumors. Tumor size was measured with caliper every 3d using the formula Volume = (a × b^2^)/2, in which a is the major axis and b is the minor axis, respectively. Mice were then randomized into different groups (Pan02-pLV-Control+IgG, Pan02-pLV-FOXP3+IgG, Pan02-pLV-FOXP3+anti-PD-L1, Pan02-pLV-FOXP3+anti-CCL5, Pan02-pLV-FOXP3+anti-PD-L1+anti-CCL5, Pan02-pLV-FOXP3+anti-PD-L1+anti-CCL5+anti-CD8) when tumors reached 70 mm^3^. Murine anti-PD-L1 antibody (Clone 10F.9G2, ) was purchased from BioXCell (clone 10F.9G2, BioXCell, UK). Subcutaneous and orthotopic tumour-bearing mice were treated by PD-L1 blockade antibody or isotype IgG via intraperitoneal (i.p.) injection. For both the subcutaneous and orthotopic tumor models, mice were treated with isotype IgG, anti-PD-L1 (200 μg/mouse, q3d, clone 10F.9G2, BioXcell) or combined with anti-CCL5 (20 μg per mouse, R&D Systems, Minneapolis, MN,USA) intraperitoneally q3d. Tumor volumes were measured every three days using a caliper. For the subcutaneous tumor model, the anti-CD8 antibody (clone 2.43, BioXCell, UK) to deplete CD8^+^ T cells was given by i.p. injection at a dose of 250 ug on days 6, 9, 15 and 21. Cell proliferation in tumor tissues was analyzed by counting anti-Ki-67 antibody positive cells in paraffin-embedded mouse tumor tissues by immunohistochemistry. For the orthotopic tumor model, C57BL/6 mice were injected with 1×10^6^ Pan02-pLV-Control-luc or Pan02-pLV-FOXP3-luc cells in Matrigel (BD Biosciences) in the tail of the pancreas. The anti-CD8 antibody (clone 2.43, BioXCell, UK) to deplete CD8^+^ T cells was given by i.p. injection at a dose of 250 ug on days5, 6, 9 and 15.Tumor growth were analysed by bioluminescent imaging. The survival time of each mouse was recorded.

Supplementary Text

**Supplemental Figure legends**

**Figure S1. The Expression of PD-L1 by Immunohistochemistry in PDAC.**

**(a)** The expression level of PD-L1 by IHC with three different cloned first antibodies (E1L3N, Cell Signaling Technology; AF156, R&D Systems; ab205921, abcam) in the same batch of pancreatic cancer tissues. (Magnification: ×200). **(b)** The correlation analysis of results of PD-L1 expression in 110 human PDAC samples between two different first antibodies. p values were calculated by Spearman’s Rank-Correlation test. R means correlation coefficient.

**Figure S2. C-FOXP3 directly activates PD-L1 transcription in PDAC.**

**(a)** Establishment and verification of stable cells lines over-expressing or down-expressing FOXP3. pLV-control and pLV-FOXP3 means lentivirus vectors as control and for over-expression of C-FOXP3; pLKO-control and pLKO-FOXP3 means lentivirus vectors as control and for knock-down of C-FOXP3. **(b, c)** The mRNA level of PD-L1 in MIA PaCa-2 and AsPC-1 cell lines were detected by real-time-PCR (mean ± SD, n=3; *P<0.05 compared with Controlβ-ACTIN was used as internal control.by Student’s t test). The proteins were detected by western blotting and flow cytometry. Quantification of PD-L1 protein levels on different pancreatic cancer cell surface. (mean ± SD, p values were by Student’s t test, **p<0.01). **(d)** Specificity of the ChIP assay. Mice PD-L1 gene, including the FOXP3 binding motif a and b (Upper). Binding of FOXP3 and PD-L1 promoter were observed at motif b. p21 was used as positive control (Down). N means negative control; P means Positive Control. **(e)** The Pan02 cells were transduced with either vector control or FOXP3 in conjunction with the luciferase reporter pGL3-PD-L1-promoter wide type WT) or pGL3-PD-L1-promoter (mutation of motif b, MUT) vectors. pGL3-p21-promoter was used as positive control. After48 h, Firefly and Renilla luciferase activities were measured using the Dual-Luciferase Reporter assay (Promega) and the ratio was determined (*P<0.05, by Student’s t test). The experiment was performed in triplicates and repeated three times with the same results.

**Figure S3. Immune Cell Isolation Efficiency Test.**

**(a, b)** Flow cytometry analysis of human and mouse CD8^+^T cell isolation. **(c)** FACS analysis of CD8^+^T cell in tumor after CD8 antibody injection in mice model.

**Figure S4. Anti-PD-L1 Antibody Effectively Reversed the Activity of CD8^+^ T cells in Co-culture System with the c-FOXP3^high^ PDAC cell lines.**

**(a)** Human (peripheral blood mononuclear cells PBMCs and mice splenocytes were isolated and stimulated with IL-2, anti-CD3 and anti-CD28 mAbs for 48 hours. Columns: Mean; Bars: Standard Deviation, n=3, p values were calculated by Student’s t test, *p<0.05, **p<0.01. pLV-control and pLV-FOXP3 means lentivirus vectors as control and for over-expression of C-FOXP3; pLKO-control and pLKO-FOXP3 means lentivirus vectors as control and for knock-down of C-FOXP3. PBMCs were co-cultured with AsPC-1-pLV-Control or AsPC-1-pLV-FOXP3 in the absence or presence of anti-PD-L1 for 18 hours. The expression of IFNγ^+^ on CD8^+^T cells were determined by flow cytometry (FACS). **(b)** Human CD8^+^ T cells were co-cultured with AsPC-1-pLV-Control or AsPC-1-pLV-FOXP3 in absence or presence of anti-PD-L1 for 18 hours. The expression of Annexin V^+^ CD8^+^T cells was determined by FACS. **(c)** Human PBMCs were co-cultured with Panc-1-pLKO-Control, Panc-1-pLKO-FOXP3, MAI PaCa-2-pLKO-Control and MAI PaCa-2-pLKO-FOXP3 in the absence or presence of anti-PD-L1 for 18 hours. The expression of IFNγ^+^ on CD8^+^T cells were determined by flow cytometry. **(d)** Human CD8^+^T cells were co-cultured with Panc-1-pLKO-Control, Panc-1-pLKO-FOXP3, MAI PaCa-2-pLKO-Control and MAI PaCa-2- pLKO-FOXP3 cell lines for 18 hours. The expression of AnnexinV^+^CD8^+^T cells was determined by flow cytometry. **(e)** Mice splenocytes were isolated from spleen of C57BL/6 mice and stimulated with IL-2, anti-CD3 and anti-CD28 mAbs for 48 hours. Then, splenocytes were co-cultured with Pan02-pLko-Control or Pan02-pLKO-FOXP3 cell lines for 18 hours. The expression of IFNγ^+^ on CD8^+^T cells were determined by FACS. **(f)** Mice CD8^+^T cells were isolated from spleen of C57BL/6 mice bearing Pan02-pLV-Control tumors and stimulated with IL-2, anti-CD3 and anti-CD28 mAbs for 48 hours. Then, CD8^+^ T cells were co-cultured with Pan02-pLKO-Control or Pan02-pLKO-FOXP3 cell lines for 18 hours. The expression of AnnexinV^+^CD8^+^T cells was determined by flow cytometry. *P<0.05, **P<0.01;

**Figure S5. Anti-PD-L1 Antibody Does not Affect PDAC Cell Lines Apoptosis and Proliferation.**

**(a)** Flow cytometry and **(b)** EDU analysis of Panc-1-pLV-Control, Panc-1-pLV-FOXP3, Pan02-pLV-Control or Pan02-pLV-FOXP3 in absence or presence of anti-PD-L1 for 24 hours. Columns: Mean; Bars: Standard Deviation, n=3, p values were calculated by Student’s t test, *p<0.05, **p<0.01. NS means not significant.

**Figure S6.** **CCL-5 and PD-L1 Antibody Combination Blockade Potently Inhibits Tumor Growth in Vivo.**

**(a)** Representative images of tumors in xenograft mice models. **(b)** Immuno-histochemical (IHC) staining of FOXP3 and Ki67 in tumor slices. Representative images were shown, Magnification: ×200. Ki67 expression level for the slides was summarized in graph. p values were calculated by one-way ANOVA tests, *P<0.05, **P<0.01; pLV-control and pLV-FOXP3 means lentivirus vectors as control and for over-expression of C-FOXP3.

Figure. S1.


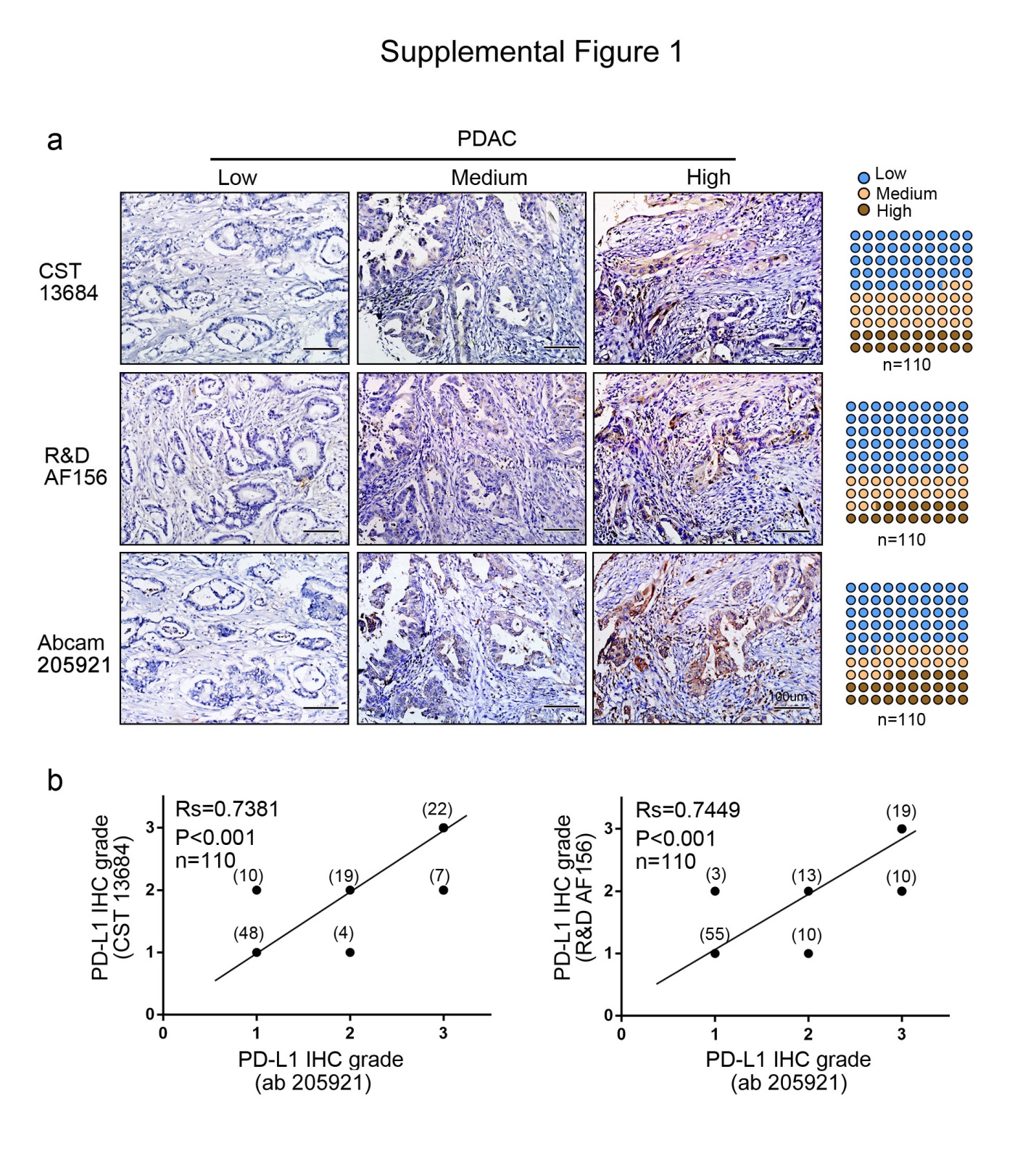


**Figure S1. The Expression of PD-L1 by Immunohistochemistry in PDAC.**

Figure. S2.

**
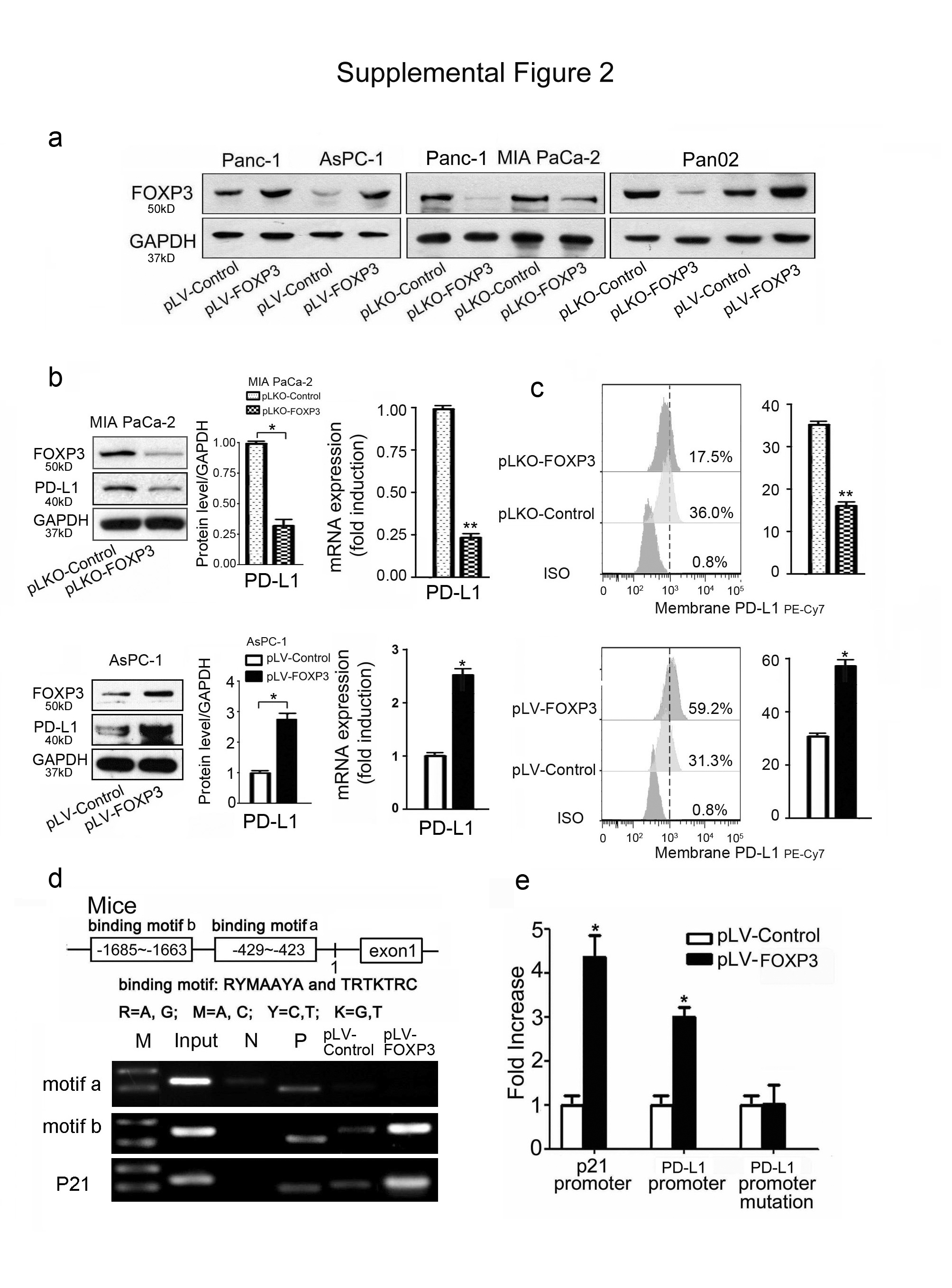
**

**Figure S2. C-FOXP3 directly activates PD-L1 transcription in PDAC.**

**Figure S3**

**
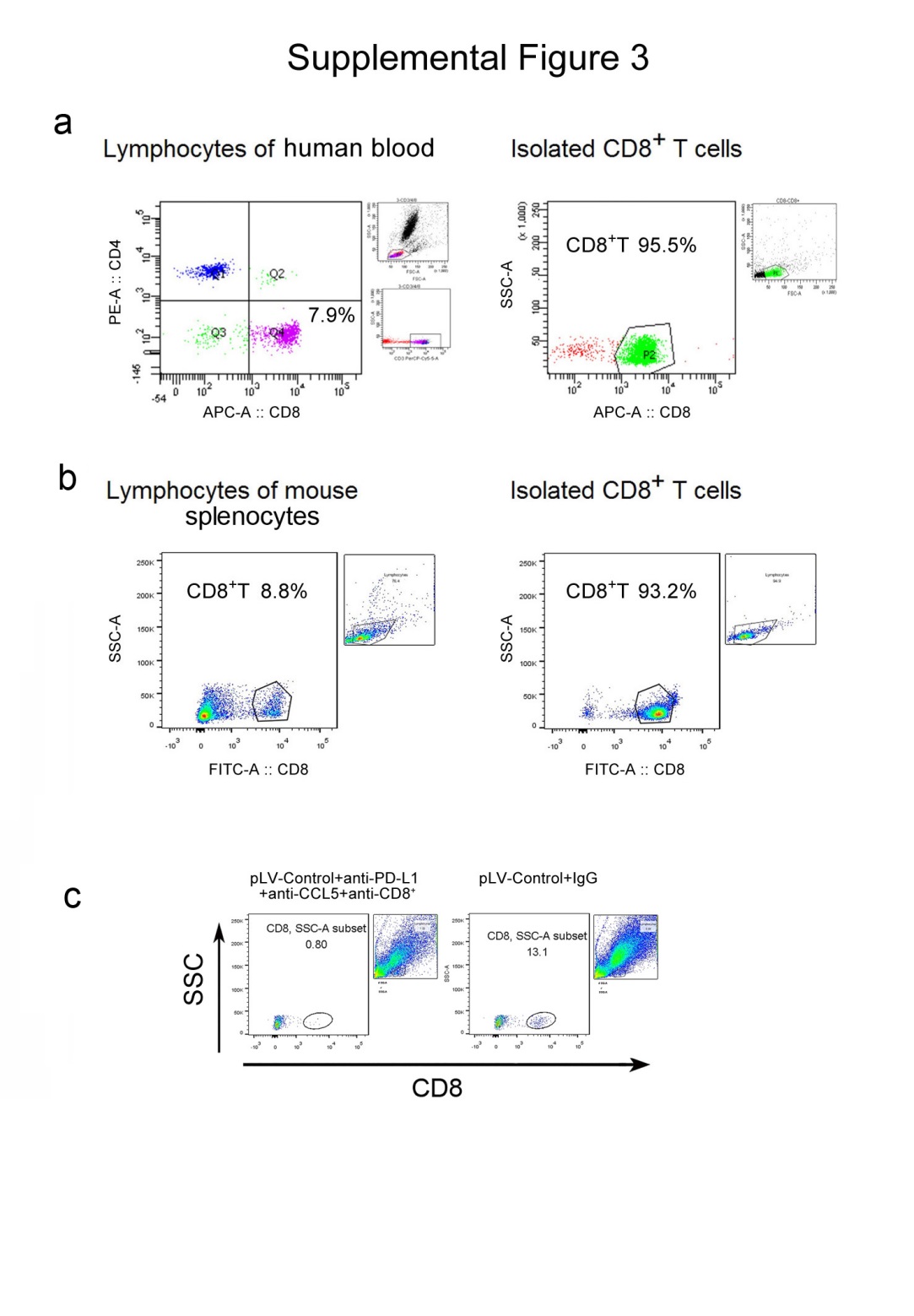
**

**Figure S3. Immune Cell Isolation Efficiency Test.**

**Figure S4**

**
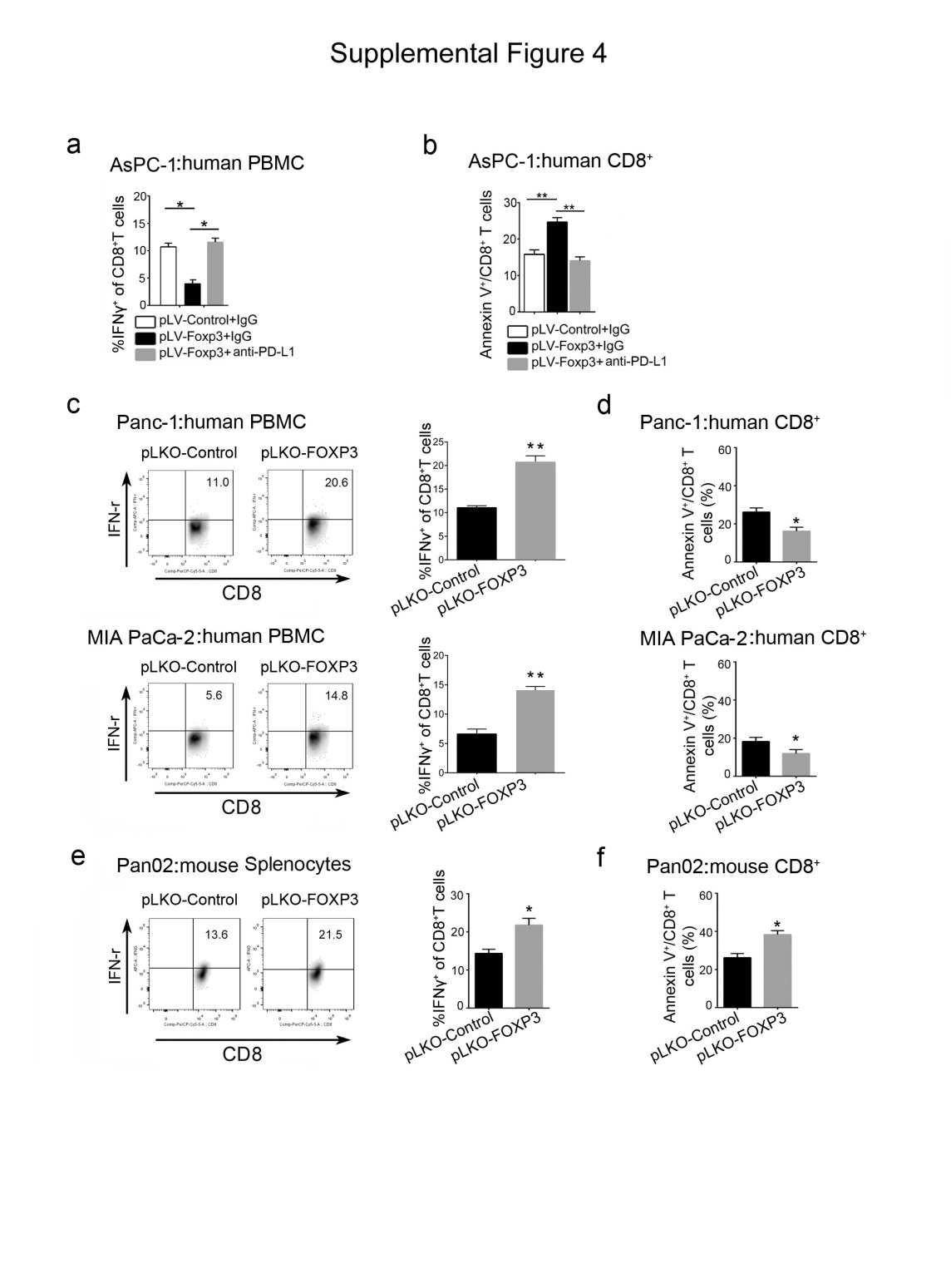
**

**Figure S4. Anti-PD-L1 Antibody Effectively Reversed the Activity of CD8^+^ T cells in Co-culture System with the c-FOXP3^high^ PDAC cell lines.**

Figure. S5


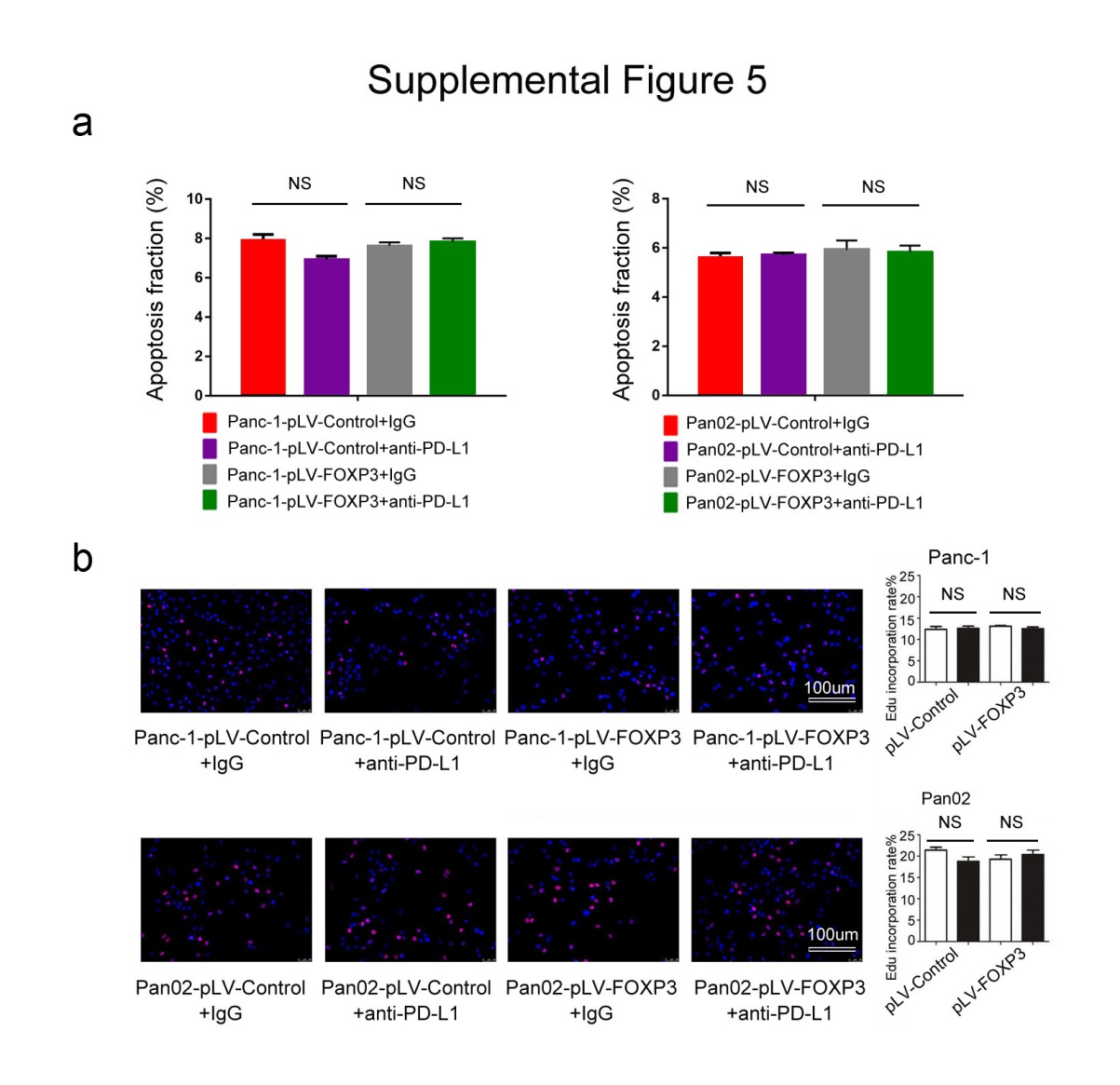


**Figure S5: Anti-PD-L1 Antibody Does not Affect PDAC Cell Lines Apoptosis and Proliferation**

**Figure. S6**


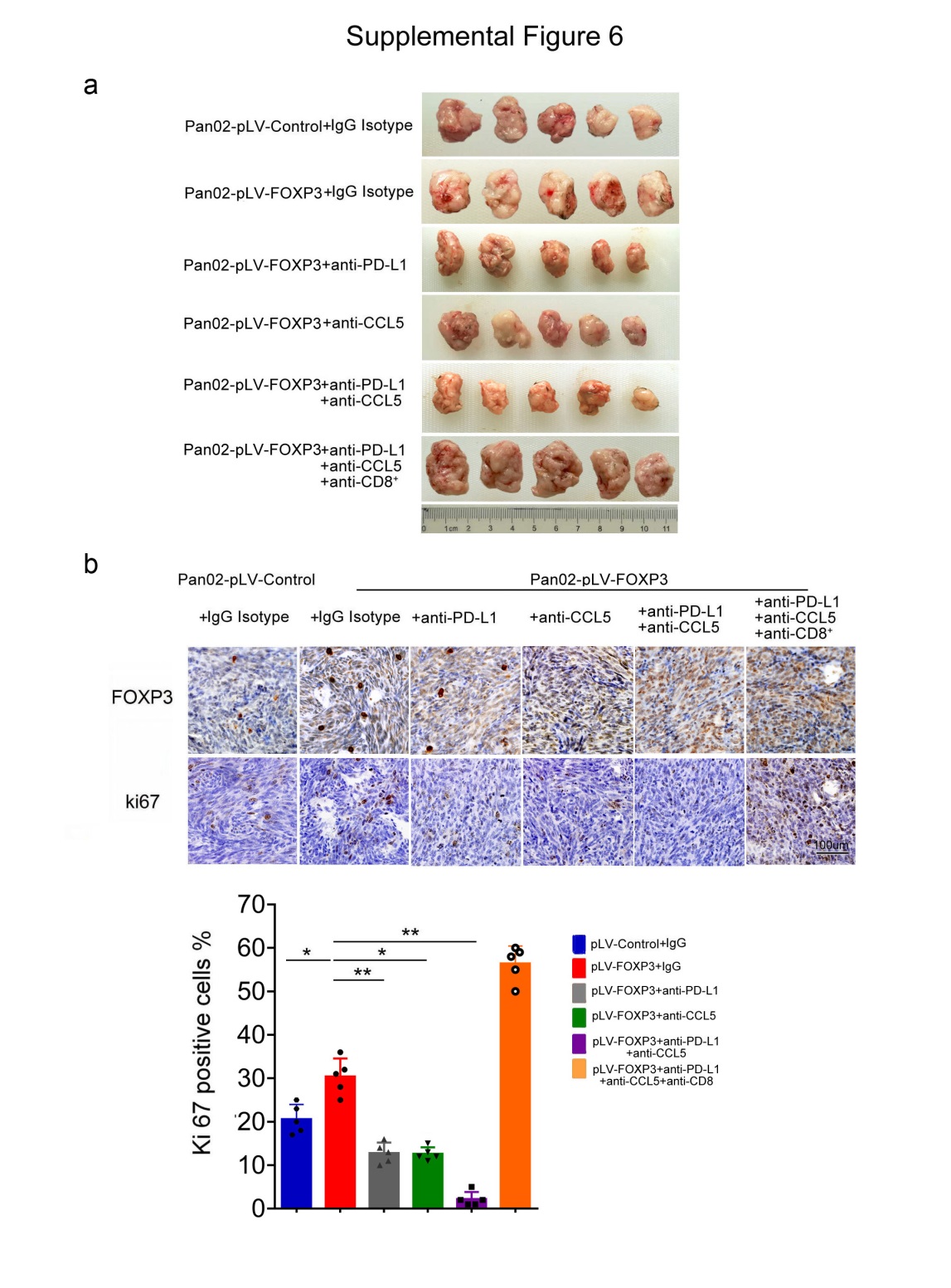


**Figure S6:** **CCL-5 and PD-L1 Antibody Combination Blockade Potently Inhibits Tumor Growth in Vivo.** **Table S1.**


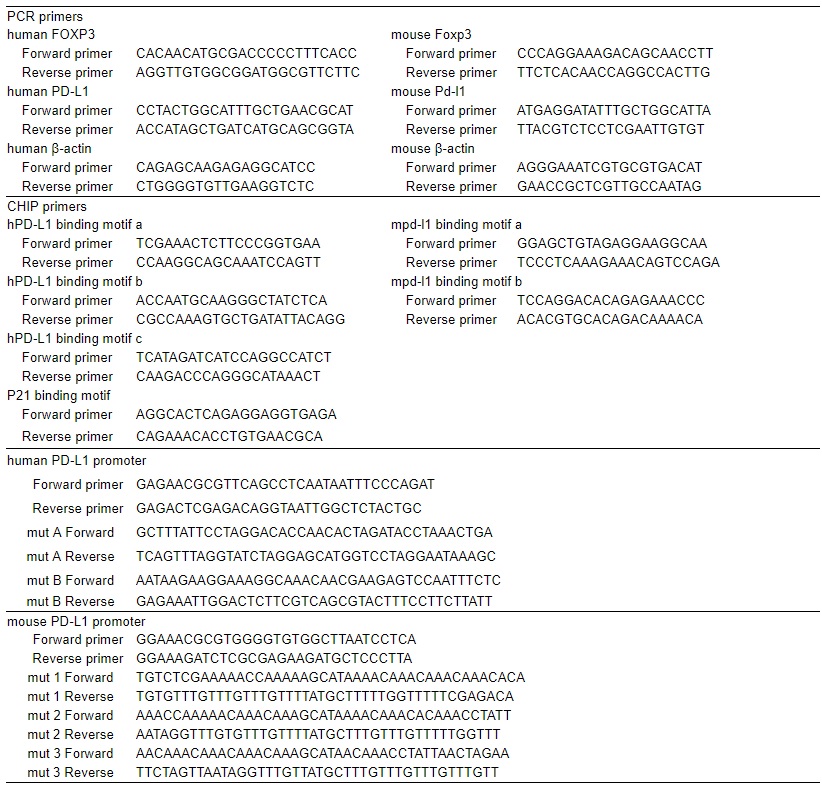


**Table S1. Primers and Oligonuceotides Sequences**

**Table S2.**

**
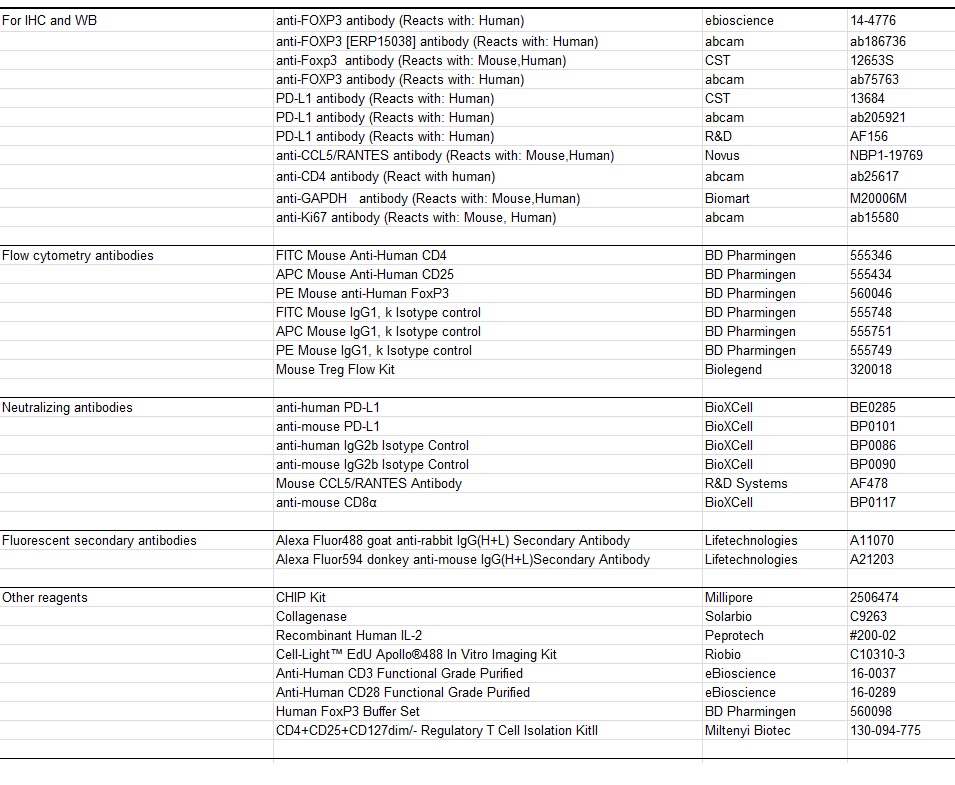
**

**Table S2. Main antibodies and reagents used in this study.**
